# New Aspects of the Kidney in the Regulation of Fibroblast Growth Factor 23 (FGF23) and Mineral Homeostasis

**DOI:** 10.3390/ijms21228810

**Published:** 2020-11-20

**Authors:** Maria L. Mace, Klaus Olgaard, Ewa Lewin

**Affiliations:** 1 Department of Nephrology, Rigshospitalet, University of Copenhagen, 2100 Copenhagen, Denmark; Klaus.oelgaard@regionh.dk (K.O.); Ewa.lewin@regionh.dk (E.L.); 2 Department of Nephrology, Herlev Hospital, University of Copenhagen, 2730 Herlev, Denmark

**Keywords:** acute kidney failure, chronic kidney failure, calcium, phosphate, klotho, PTH, calcitriol, activin A, CKD-MBD, circadian rhythm, bone, crosstalk

## Abstract

The bone-derived hormone fibroblast growth factor 23 (FGF23) acts in concert with parathyroid hormone (PTH) and the active vitamin D metabolite calcitriol in the regulation of calcium (Ca) and phosphate (P) homeostasis. More factors are being identified to regulate FGF23 levels and the endocrine loops between the three hormones. The present review summarizes the complex regulation of FGF23 and the disturbed FGF23/Klotho system in chronic kidney disease (CKD). In addition to the reduced ability of the injured kidney to regulate plasma levels of FGF23, several CKD-related factors have been shown to stimulate FGF23 production. The high circulating FGF23 levels have detrimental effects on erythropoiesis, the cardio-vascular system and the immune system, all contributing to the disturbed system biology in CKD. Moreover, new factors secreted by the injured kidney and the uremic calcified vasculature play a role in the mineral and bone disorder in CKD and create a vicious pathological crosstalk.

## 1. Introduction

The endocrine network maintaining calcium and phosphate homeostasis involves a complex interplay between several hormones that exert their function in bone, kidneys and intestine. The plasma concentration of ionized calcium (Ca^2+^) is tightly regulated, whereas the phosphate concentration varies slightly. The discovery of the bone-derived hormone fibroblast growth factor 23 (FGF23) in 2000 led to a new understanding of the mineral homeostasis [1]. It also illustrated that bone is not merely a structural component and a reservoir for minerals, but also an active player in the complex endocrine system maintaining the mineral balance. Later, FGF23 was identified as a fundamental component of the mineral and bone disorder in chronic kidney disease (CKD) and has been the focus for extensive research [2]. The present review summarizes the regulation and function of FGF23 and the disrupted FGF23 and mineral homeostasis in CKD.

## 2. FGF23 in Normal Physiology 

### 2.1. Secretion and Metabolism of FGF23

Fibroblast growth factors (FGFs) belong to the superfamily of genes exerting pleiotropic effects in a broad range of biological processes via activation of the FGF receptor tyrosine kinase (FGFR). FGF23 is a member of the endocrine FGF family. It is primarily secreted from osteocytes and osteoblasts in bone and acts on the kidney to promote phosphaturia, regulates the synthesis and degradation of the active vitamin D metabolite calcitriol and urinary calcium excretion [3]. FGF23 is a 32-kDa glycoprotein containing a proteolytic site. The modification of FGF23 protein by O-glycosylation and phosphorylation controls the proteolytic cleavage. The O-glycosylation of FGF23 by N-acetylgalactosaminyltransferase 3 (GALNT3) prevents the cleavage, leading to an increase in circulating intact FGF23 [4]. The full-length protein is biologically active and its cleavage in vivo results in generation of a C-terminal and a N-terminal fragment. The full-length and the C-terminal FGF23 are detectable in the circulation. The C-terminal peptide retains the ability to bind to the FGFR/αKlotho complex but without inducing signaling [5]. Thus, potentially, the C-terminal domain functions as a naturally occurring competitive antagonist. There are four FGFRs: FGFR1–4. FGF23 preferentially binds to FGFR1c, 3c and 4 isoforms, and the presence of the obligate co-receptor αKlotho (Klotho) is required for the high affinity binding of FGF23 to FGFR [6,7]. Due to the universal expression of FGFRs, tissue specificity of the action of FGF23 is conferred to the presence of Klotho. Kidneys, parathyroid glands and the brain are the primary organs with abundant expression of Klotho [8,9]. 

In bone, FGF23 biosynthesis is regulated by differential factors and pathways. High plasma phosphate level on a long-term basis is a positive regulator of FGF23. Acute intravenous administration of phosphate is, however, not affecting plasma FGF23 levels [10,11,12]. The mechanism involved in sensing of phosphate by osteocytes is not fully understood, however, new putative sensors and pathways are emerging. The type III phosphate cotransporter PiT2 has been proposed to mediate the extracellular phosphate dependent regulation of FGF23 production. PiT2 deletion in mice resulted in blunted FGF23 response to high or low phosphate diet [13]. FGFR1c has also been proposed as a phosphate sensing molecule in bone [14]. Genetic and pharmacologic interventions have shown that activation of FGFR1 induces FGF23 synthesis and inhibition of FGFR signaling attenuates FGF23 production [12,15,16,17,18,19,20]. FGFR1c was recently shown to be activated by high dietary phosphate in a ligand-independent manner through phosphorylation of the FGFR substrate 2α and ERK. Downstream, this signaling pathway regulated the expression of GALNT3 involved in the posttranslational modification of FGF23 protein [14]. High extracellular phosphate has also in osteoblastic cells in vitro been demonstrated to enhance the expression of FGF23 through stimulation of the nicotinamide adenine dinucleotide phosphate (NADPH) oxidase induced reactive oxygen species (ROS) production and MEK-ERK pathway [21]. 

Crosstalk between FGF23, parathyroid hormone (PTH) and calcitriol is of great importance for controlling mineral homeostasis. PTH is an important positive regulator of FGF23. Activation of PTH1R by PTH induces cAMP/PKA and the transcription factor NURR1, this turns on transcription of FGF23 [22]. Constitutive activation of PTHR in transgenic mice increased FGF23 synthesis in osteocytes via a Wnt-dependent mechanism [23]. Calcitriol is, via its binding to the vitamin D receptor (VDR)/RXR complex and to vitamin D responsive element (VDRE) on the promoter of FGF23, a potent stimulator of FGF23 expression [24]. Both systemic calcitriol and local production of calcitriol in osteoblasts are positive transcriptional regulators of FGF23 [25]. Erythropoietin (EPO), hypoxia inducible factors (HIFs), and various inflammatory stimuli are all involved in FGF23 regulation, related to pathophysiological conditions [26].

**Figure 1:**
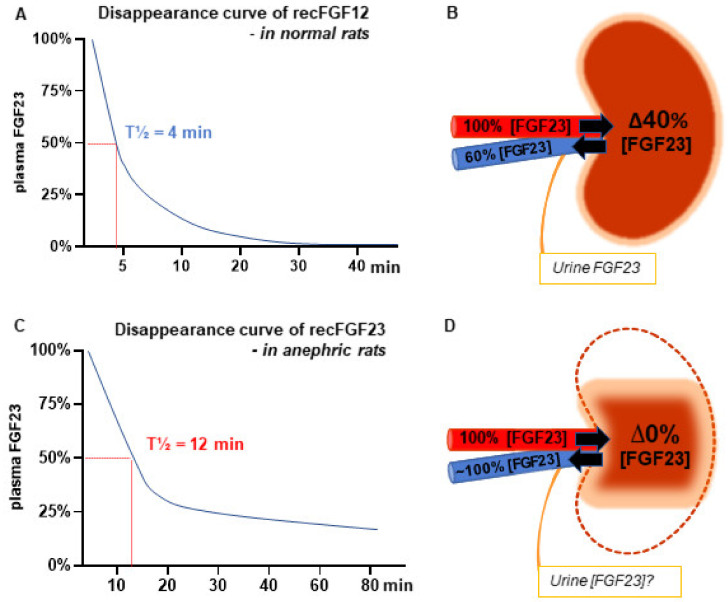
The key role of the kidney in regulating the plasma levels of fibroblast growth factor 23 (FGF23) (**A**) Disappearance curve of recombinant FGF23 (recFGF23) in normal rats. Rats were treated with the pan fibroblast growth factors (FGF) receptor tyrosine kinase (FGFR) inhibitor PD173074 to suppress FGF23 production in bone, resulting in negligible plasma levels of FGF23 prior to iv. administration of the recFGF23. The clearance of recFGF23 fitted a first order elimination and FGF23′s half-life (T½) was calculated as 4 min. (**B**) Measurement of intact FGF23 in the renal artery and vein demonstrates a high extraction ratio of 40%. (**C**) RecFGF23 was administered to bilateral nephrectomized (anephric) rats after suppression of endogenous FGF23 levels. The recFGF23 had a prolonged clearance and T½ was increased to 12 min in the anephric rats. (**D**) Measurement of intact FGF23 in the renal artery and vein in the kidney rudiment of the 5/6 nephrectomy model after 8 weeks of uremia. Similar FGF23 concentrations were found, illustrating that the injured kidney loses its ability to regulate plasma levels of FGF23. One of the key mechanisms behind the increase in plasma levels of FGF23 in kidney disease [12,19].

We estimated the half-life of FGF23 by measuring the disappearance of exogenous recombinant FGF23 in the rat and found a rather short half-life of 4 min (Figure 1A) [12]. To investigate the role of the kidney in the regulation of FGF23, we measured the concentration of FGF23 in the renal artery and renal vein and found a significant renal extraction of the hormone (Figure 1B). Commercially available ELISAs could not measure FGF23 in urine and we were therefore not able to calculate the renal clearance of the hormone [12]. Other groups detected FGF23 in urine samples using other, however, non-quantitative methods [2,27,28,29]. A later study in humans demonstrated a similar renal extraction ratio of FGF23, which was similar to that of creatinine [30]. 

### 2.2. Circadian Rhythm of Plasma FGF23

**Figure 2:**
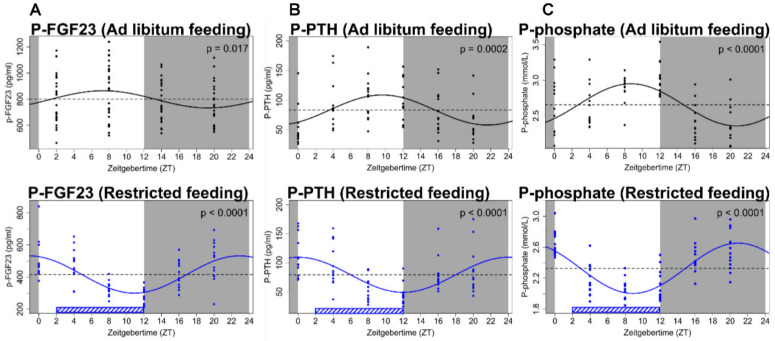
Circadian rhythm of FGF23 and mineral parameters. (**A**) Plasma levels of FGF23 show significant diurnal variation. Phase of fluctuation was complete reversed by feeding restricted to the inactive period (lower diagram). (**B**,**C**) Parathyroid hormone (PTH) and phosphate levels varied around the clock. Their acrophase and nadir were consistently shifted to the opposite value by the restricted feeding intervention. Data are fitted by cosinor regression and resulting *p*-values are shown, *p* < 0.05 is considered significant. Grey areas indicate dark period and white areas indicate light period. The figure has been published in Kidney International, Egstrand et al., 2020 [32].

The circadian rhythms in hormonal levels have a systemic impact on all aspects of physiology. Results from our laboratory showed the existence of a circadian rhythm of plasma FGF23 levels (Figure 2A) [31,32]. The physiological importance of FGF23 rhythmicity remains, however, to be established. Similarly, it remains to be established which specific input that determines the phase of the circadian rhythm of plasma FGF23. 

In mammals the master pacemaker of circadian rhythmicity is in the hypothalamic suprachiasmatic nucleus (SCN). The SCN receives light cues about day and night and it is accordingly coordinating the peripheral and central clocks via neuronal, hormonal and metabolic signaling pathways [33]. In addition to this central pacemaker, a molecular clock machinery has been demonstrated in a number of peripheral tissues. Although light is the dominant environmental cue for SCN, some peripheral tissues are sensitive to feeding and fasting [34]. The kidneys have a robust internal molecular circadian clock, and many genes that determine renal function are expressed in a circadian manner [35,36]. As such, the excretion of FGF23 by the kidney might potentially exhibit a circadian pattern affecting plasma levels. 

Bone remodeling is a complex process by which old bone is removed and replaced by new bone, requiring interaction between different bone cells, a process which to a large extent is coordinated by osteocytes [37]. The diurnal variation in bone turnover markers and the demonstration of an internal bone circadian clock may indicate that circadian rhythmicity is an important aspect of bone homeostasis [38]. The circadian rhythm of plasma FGF23 could potentially be the result of a local circadian regulation of secretion or degradation of FGF23 in bone. Both direct and indirect effects of pituitary hormones on bone remodeling have been demonstrated [39]. Nevertheless, it is not known whether the synthesis of FGF23 is controlled by a superior “hypothalamic–pituitary axis” as seen in most other endocrine tissues. Interestingly, an association between Klotho and growth hormone has been proposed to exist in acromegaly [40]. Still, plasma levels of Klotho do not express circadian rhythmicity, as demonstrated by our group [31]. 

Furthermore, plasma phosphate and PTH have circadian rhythms that depend on the feeding pattern [32]. This phenomenon should be taken into consideration when interpreting the circulating levels of FGF23. We have recently demonstrated that changing the feeding pattern shifted the phase of the circadian rhythm of plasma phosphate, PTH and FGF23. Rats were acclimatized to standard conditions of 12:12h light:dark cycle and ad libitum feeding for 2 weeks and so blood samples were obtained at a 4 h interval for 24 h. Feeding was then restricted to the light phase (inactive phase in nocturnal rodents) for 4 weeks and the blood sampling was repeated. Feeding restricted to the inactive phase markedly shifted the phase of plasma FGF23 together with that of plasma PTH and phosphate, still exhibiting significant circadian rhythms but at a reverse-phase pattern that mirrored the one of rats with continuous access to feeding (Figure 2) [32]. As such, the intestine–bone axis might have an impact on the circadian rhythmicity of plasma FGF23 levels and on the circadian rhythmicity of hormones and factors that are regulating FGF23 secretion on long term basis.

### 2.3. FGF23, Klotho and Kidney 

In the kidney, FGF23 binds to FGFR. The obligate co-receptor Klotho is expressed in the distal tubules and to less extend in the proximal renal tubules [8,41]. Inhibition of phosphate reabsorption in the proximal renal tubules by FGF23 is mediated through activation of ERK1/2 and SGK1, leading to phosphorylation of NHERF-1, which induces internalization and degradation of the sodium-phosphate co-transporter NaPi2a [42]. Less availability of NaPi2a on the apical brush border membrane results in a decrease in active phosphate reabsorbed from urine. FGFR1 is the main receptor responsible for the FGF23 mediated phosphaturia. FGFR4 also plays some, albeit a minor role [43,44]. 

It is probably the transmembrane Klotho that is expressed in small quantities in the proximal renal tubules, which is the co-receptor responsive for FGF23–FGFR interaction in the kidney. It has also been proposed that a circulating soluble Klotho fragment acts as a hormone on the proximal tubule, causing endocytosis of the NaPi co-transporters [41,45]. Klotho is a single-pass type I membrane protein of approximately 130 kDa with a short cytoplasmic domain and a large extracellular domain composed of two tandem repeats Kl1 and Kl2. The extracellular domain of Klotho is cleaved by the membrane anchored proteases ADAM 10 and 17, shed on the cell surface and can be detected in blood [46,47]. Transepithelial transport of soluble Klotho through the basolateral membrane, the cytosol and ultimately reaching the brush-border membrane has been shown [41].

In the distal convoluted tubule FGF23 increases calcium reabsorption. FGF23 is acting via the FGFR–Klotho complex and activation of ERK1/2, SGK1 and WNK4 upregulates expression of the calcium-selective channel protein TRPV5 at the luminal membrane and hereby reduces renal calcium excretion [48]. The phosphaturic and calcium conserving renal effects of FGF23 are similar to those of the other calcium and phosphate regulating hormone PTH. In contrast, the function of FGF23 signaling involved in the regulation of the key enzyme responsible for calcitriol production, the 1α-hydroxylase (*CYP27B1*), is opposite to that of PTH. The 1α-hydroxylase is expressed in the proximal renal tubule and is regulated by both FGF23, PTH and calcitriol itself. FGF23 suppresses the 1α-hydroxylase expression and activity [49,50]. A principal role of FGF23 in the regulation of 1α-hydroxylase has been demonstrated in Klotho and FGF23 hypomorphic mice, these have an inappropriately high 1α-hydroxylase expression, despite hypercalcemia and suppressed PTH [8,49]. In addition, FGF23 may directly or indirectly increase the synthesis of the catabolic enzyme 24-hydroxylase and hereby further reduce the activity of calcitriol [49,51].

### 2.4. FGF23 Regulates PTH Production

The direct interplay of FGF23 and PTH takes place at the level of bone and in the parathyroid glands. In bone, PTH stimulates biosynthesis of FGF23 via activation of the orphan nuclear receptor Nurr1 [22]. The parathyroid gland expresses both FGFRs and Klotho and it is a target for FGF23 action. FGF23 inhibits PTH biosynthesis and secretion via Klotho-FGFR activation of the MAPK/ERK1/2 signaling pathway [9]. Furthermore, FGF23 increases the parathyroid expression of the calcium sensing receptor (CaSR) and VDR, both contributing to the suppression of PTH [52]. Surprisingly, it has also been shown that FGF23 is positively associated with parathyroid cell proliferation via activation of Klotho-FGFR signaling [53]. The parathyroid gland loses rapidly its responsiveness to extracellular calcium ex vivo and a functional parathyroid cell line has not yet been established [54]. We examined in vivo the impact of FGF23 on the Ca^2+^/PTH relationship during normocalcemia and acute hypocalcemia in the rat and demonstrated that FGF23 has an inhibitory tonus on PTH secretion, when plasma Ca^2+^ is within the normal range. However, if plasma Ca^2+^ is low and increased PTH secretion is needed in order to restore Ca^2+^ levels, this inhibitory effect of FGF23 on PTH secretion is abolished. Furthermore, the same experimental in vivo study showed that the suppressive tonus of FGF23 in normocalcemia was mediated through the FGFR [20].

The role of parathyroid Klotho remains controversial. Klotho has been proposed to modulate the parathyroid Na^+^/K^+^-ATPase activity via interaction with the α-1-subunit and hereby causing an increased abundance of Na^+^/K^+^-ATPase in the plasma membrane [55]. This results in an increased electrochemical gradient, which may promote PTH secretion in hypocalcemia. In contrast to this hypothesis, our group has later demonstrated that blocking the Na^+^/K^+^-ATPase by ouabain did not affect the PTH secretory response to hypocalcemia [56]. Our results underscored that parathyroid Klotho alters the glandular sensitivity to calcium via regulation of Na^+^/K^+^-ATPase activity. Furthermore, it has since been demonstrated that parathyroid specific deletion of Klotho did not alter the PTH acute response to changes in extracellular calcium or FGF23. Additionally, in the absence of parathyroid Klotho, the calcineurin-NFAT pathway was found to mediate the suppression of PTH secretion by FGF23 [57]. The mechanisms of FGF23 signaling in the parathyroid gland have now expanded into two principal pathways, involving Klotho-FGFR activation and Klotho independent calcineurin activation, respectively [57].

## 3. FGF23 and the Disturbed Mineral Balance in Kidney Disease

A central complication in CKD is the mineral and bone disorder (CKD-MBD), a complex systemic disorder characterized by disturbances in calcium and phosphate balance, plasma levels of PTH and calcitriol along with changes in bone morphology, bone density and remodeling activity. Development of calcification in soft tissue especially the arteries and heart valves are also part of this complex syndrome [58]. Discovery of the FGF23/Klotho system has improved the understanding of the CKD-MBD as it plays a central role in the disorder. Plasma levels of FGF23 increase early in kidney disease before derangements in plasma phosphate and PTH [59]. Around this time, it is thought that Klotho is downregulated in the kidney. However, due to poor Klotho antibodies the possible changes in circulating Klotho have not been completely clarified [47]. Before uncovering the FGF23/Klotho system, it was thought that the reduced kidney mass failed to produce sufficient calcitriol levels. However, the decrease in calcitriol might also be attributed to the inhibitory effect of FGF23. Subsequently, secondary hyperparathyroidism develops due to low calcitriol, hypocalcemia and hyperphosphatemia, when the phosphate load exceeds the tubular excretion capacity [60].

### 3.1. Role of the Kidney in Secretion and Metabolism of FGF23

Patients with CKD have severely increased plasma levels of FGF23 and deficiency of Klotho. Epidemiological studies have repeatedly reported that FGF23 is an important factor associated with increased risk of progression of kidney disease, loss of kidney graft function, cardiovascular disease and mortality [61,62,63,64,65,66].

The mechanisms behind this early increase in FGF23 are not completely understood. It has been proposed to be an early physiological mechanism in order to increase phosphate excretion in the functional nephrons [67]. Whether the downregulation of FGF23′s co-receptor Klotho has an effect on the FGF23 increase is not known. It is not clear if changes in Klotho expression precede or follow the initial FGF23 increase [68]. Plasma levels of FGF23 increase progressively as kidney function declines and have been correlated to estimated glomerular filtration rate, indicating that the kidney plays an important role in the regulation of FGF23 [2,69,70,71]. A rise in plasma levels of FGF23 has also been found in acute kidney injury (AKI) [72,73,74].

Therefore, we investigated the role of the kidney in the regulation of FGF23 in experimental models in the rat [12,19]. Removal of the two kidneys resulted in a 2.5 doubling in the plasma levels of FGF23 within 15 min, illustrating the importance of normal kidney function in maintaining the plasma levels of FGF23. Removal of one kidney also caused a significant rise in FGF23 reaching a level right in between the levels of the anephric and normal control rats, further illustrating the importance of kidney mass. To study whether the rise in FGF23 was the intact FGF23 protein or FGF23 fragments, we calculated the ratio between them and found it to be significantly higher than the corresponding value in normal rats [12]. So, the kidney regulates the circulating biological active protein. Similar results have been found in mice with AKI [74]. These results are in accordance to observations in humans showing that circulating FGF23 in patients with kidney failure mainly consists of the intact molecule [75]. To examine the potential impact on FGF23 clearance, we administered recombinant FGF23 to anephric rats and demonstrated a prolonged disappearance curve and FGF23 half-life was increased from 4 to 12 min (Figure 2C) [12]. These results illustrate the importance of renal extraction of the hormone. As the intact FGF23 protein is 32 kDa (28 kDa non-glycosylated form) it could be filtered in the glomerulus and so its gradual increase in plasma levels that follows the inverse decrease in glomerular filtration rate, as reported in epidemiological studies, could be explained hereby. 

In a model of chronic kidney disease, the 5/6 nephrectomized rat, we have repeatedly measured severely increased plasma levels of FGF23 [76,77,78,79]. Similarly, to the findings in CKD patients. Interestingly, we demonstrated no significant renal extraction of the hormone in this model of CKD, as the FGF23 concentration in the renal artery and vein was the same (Figure 1D). These results illustrate the reduced ability of the injured kidney to regulate plasma levels of FGF23 in CKD and underline one of the key mechanisms behind the increase in plasma levels of FGF23 in kidney disease [19]. 

### 3.2. FGF23 Regulation in Kidney Disease

FGF23 plasma levels increase early in CKD progression [59]. Besides the impaired renal extraction of the intact FGF23 molecule and the downregulation of Klotho expression in the kidney, several other factors in the uremic condition affect FGF23 balance. The phosphate load in CKD seems to have effect on FGF23 expression and secretion. Our group and others have shown that a low phosphate diet significantly suppresses the plasma levels of FGF23 in CKD models [31,80,81]. Manipulating phosphate load in patients has also been reported to reduce circulating FGF23 yet not consistently [82,83,84,85]. Calcitriol continues to stimulate FGF23 expression in uremia and so treatment with active vitamin D analogs in CKD patients may not only target PTH levels but also unwanted FGF23 expression in bone [86]. 

Regulation through the bone FGFR maintains to be an important regulator of FGF23 in kidney insufficiency [12,18,19]. As shown by experimental studies by our group the FGFR inhibitor PD173074 completely turned off FGF23 gene in bone of normal and CKD rats [12,19]. Other complications to CKD such as metabolic acidosis and inflammation stimulates FGF23 expression [87,88]. Additionally, disturbances in erythropoiesis in kidney disease such as anemia, deficiency and supplementation of erythropoietin and iron, all have an effect on FGF23 synthesis and degradation resulting in increased plasma levels of FGF23 [88,89,90,91]. 

**Figure 3:**
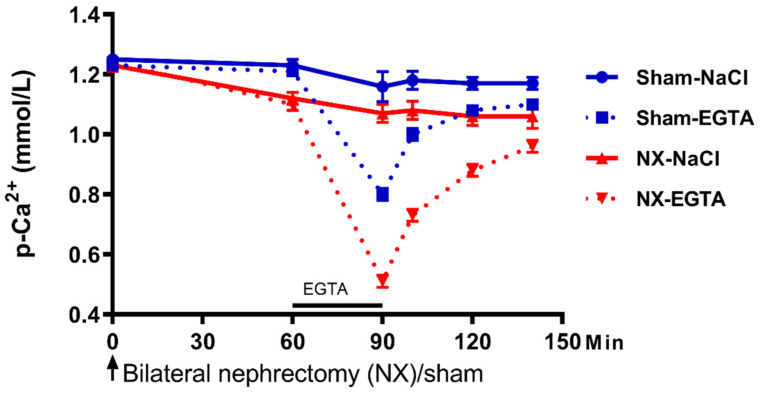
The role of the kidney in setting the set point for calcium sensing. Rats underwent bilateral nephrectomy (NX) or sham surgery. Removal of the kidneys resulted in a significant drop in plasma Ca^2+^, illustrating a role of the kidney in the setting of Ca^2+^ set point. The calcium chelator EGTA was infused via the femoral vein and it lowered plasma Ca^2+^ in both groups, however, more pronounced in the bilateral nephrectomized rats. The concentration of plasma Ca^2+^ remained, at all time points, significantly lower in these rats during recovery from hypocalcemia (*p*  <  0.01). Data are presented as mean ± SEM. The figure has been published in BMC Nephrol, Nordholm et al., 2015 [96].

The bone disease in CKD ranges from adynamic bone disease to a high turnover condition including varied levels of mineralization defect and disturbed bone volume. The type of bone disorder in CKD has not been related to FGF23 expression. One similarity between the very different bone disorders in renal osteodystrophy is the impaired function of the skeleton to buffer excess calcium and phosphate. The interchangeable pool of calcium occurs on the bone surface and its function is separate from the remodeling activity [92]. Ca^2+^ is sensed by the CaSR which is expressed in bone. However, the molecular mechanism behind the rapid influx/efflux of Ca^2+^ in bone is not fully understood [93]. The concept of how extracellular concentration of phosphate is sensed by the cell is still evolving. Besides the earlier proposed role of phosphates transporters and FGFR1, a recent study examining the crystal model of the structure of the CaSR revealed sites for phosphate binding [94]. Phosphate alters the receptor to the inactive state and thereby triggers PTH secretion [95]. We studied the effect of acute hyperphosphatemia and hypercalcemia in rats after removal of the two kidneys, however, we found no difference in the buffer capacity of normal and anephric rats for the two minerals despite significantly increased FGF23 circulating levels in the anephric rats [12]. In another study from our group bilateral nephrectomy rapidly decreased the plasma concentration of Ca^2+^ and the Ca^2+^ set point on bone surface. Inducing acute hypocalcemia by short lasting EGTA infusion in normal rats lowered plasma Ca^2+^ significantly. Then the EGTA infusion was stopped and subsequently a rapid increase in plasma Ca^2+^ took place (within 10 min) with further recovery to basal levels. EGTA infusion in anephric rats caused lower calcium levels and recovery to a lower levels of plasma Ca^2+^ (Figure 3). Plasma phosphate levels were, on the contrary, stable [96]. 

Even though calcium and phosphate share interrelated regulatory pathways, the bone’s ability to buffer calcium and phosphate is dissociated from each other. Further studies are needed to improve the understanding of the role of the kidney in the minute-to-minute regulation of calcium and phosphate. In previous studies, we demonstrated that PTH, calcitriol and calcitonin are not required for the minute-to-minute recovery from acute hypocalcemia but the hormones are setting the set point for upregulation of calcium levels and hereby having an additional impact on mineral homeostasis [97,98,99,100]. How the set point for phosphate is regulated requires further studies. 

### 3.3. Kidney Insufficiency Causes Disturbed Circadian Rhythm of Plasma FGF23, Phosphate and Hormones Involved in the Mineral Homeostasis 

We examined the circadian rhythm of mineral parameters in normal rats and 5/6 nephrectomized (CKD) rats fed a low, normal or high phosphate diet [31]. Plasma concentration of Ca^2+^ was the same around the clock in all CKD groups. On the contrary, plasma levels of phosphate were lowest in the active phase (early morning) and highest in the inactive phase (afternoon) in normal rats. As rats are nocturnal animals, the corresponding value in human would be opposite to the time point. This diurnal cycle was maintained but shifted in time in all CKD rats. The circadian rhythm of plasma FGF23 was disturbed including a shifted acrophase in CKD rats fed a high phosphate diet or standard diet. FGF23 circadian rhythmicity was completely abolished in CKD rats fed a low phosphate diet, which also remarkedly lowered circulating FGF23 in these rats. Plasma levels of Klotho were similar during day and night both in normal and uremic rats. Circulating PTH levels changed significantly during the 24 h clock with lowest levels in the active phase (early morning) and in the highest levels inactive phase (midday). This diurnal variation was completely abolished in CKD rats fed a high phosphate diet. Low phosphate diet preserved significant circadian rhythm although acrophases were shifted [31]. Disturbed circadian rhythmicity is a feature of CKD-MBD, deserving further investigations [34]. A disturbed circadian clock has been linked to several diseases such as cardiovascular disease and cancer [101,102].

We further examined the circadian clock operating the parathyroid gland in normal and in CKD rats suffering from secondary hyperparathyroidism. It was shown that an internal molecular circadian clock operates in the parathyroids and that a circadian rhythmicity of genes, regulating cell cycle was demonstrated [32]. In the hyperplastic glands, key genes of the circadian clock machinery were deregulated including shifted or abolished rhythmicity over the 24 h [32]. Parathyroid tissue from patients with primary or secondary hyperparathyroidism has also shown deregulation of clock genes [103]. Still, the pathophysiological importance is not clarified but our results point toward a potential contribution of a disturbed circadian clock in the development of parathyroid hyperplasia in CKD.

### 3.4. FGF23/Klotho System in Kidney Failure 

Early in kidney injury the expression of Klotho is downregulated. The precise mechanisms have not been completely clarified. A rapid unilateral decline in kidney Klotho expression was demonstrated in a model of unilateral obstructive nephropathy (UUO), despite the condition is not a state of uremia [104]. Its downregulation is associated with upregulation of factors related to inflammation and fibrosis in the kidney. The contribution of reduced Klotho expression in the kidney to initiate and maintain the FGF23 upregulation in kidney insufficiency is not known. The increased circulating FGF23 is still able to execute its function in the injured kidney and thereby maintains a normal plasma level of phosphate until late stage of kidney disease. However, the important counterregulatory role of calcitriol activity in normal physiology is altered in kidney disease to an inappropriate downregulation of calcitriol, contributing to the development of secondary hyperparathyroidism. Still, the phosphaturic function of FGF23 is highly important in maintaining a normal plasma level of phosphate in CKD as shown in an experimental study on 5/6 nephrectomized rats treated with the FGF23 antibody. Although the FGF23 antibody treatment normalized levels of calcium, calcitriol, PTH and bone markers, the rats had increased mortality due to cardiovascular events related to hyperphosphatemia [105]. 

We and others have demonstrated that induction of FGF23 expression in acute and chronic injured kidney tissue regardless of animal model [19,106,107,108]. There are conflicting results on the intrarenal localization of FGF23, initially proposed to be expressed in the tubular cells. This is probably due to challenging problem of FGF23 antibodies specificity. We have shown by in situ hybridization, the location of FGF23 to be exclusively expressed by cells in the interstitial space. The kidney’s expression of FGF23 is not regulated by PTH or FGFR signaling. Additionally, it has no effect on the expression of Klotho and the phosphate co-transporters in the kidney. The kidney-derived FGF23 does not contribute to circulating FGF23 (Figure 1D) [19]. The study by Smith et al. showed that kidney-derived FGF23 induced pro-fibrotic signaling via activation of the TGF-β pathway [108]. Therefore, autocrine/paracrine functions of kidney-derived FGF23 in inflammation or fibrosis processes seem plausible. However, whether it has a positive or negative effect on kidney disease progression is yet to be clarified. Expression of FGF23 has been shown in other injured organs and tumors [109,110,111].

Due to the very high concentration of FGF23 in plasma measured in CKD, it is thought to activate FGFRs independently of Klotho and several off-target effects of FGF23 have been reported. It has been shown in experimental CKD models that FGF23 activates FGFR4 and the downstream calcineurin-NFAT pathway in cardiomyocytes and induces left ventricular hypertrophy [112,113]—a common complication seen in CKD patients. It seems that something in the uremic environment may prime the cardiomyocytes to FGF23 signaling, since left ventricular function was found to be normal in patients suffering from increased FGF23 due to genetic causes and in an animal model of x-linked hypophosphatemia [114,115]. Although genetic disease is associated with disturbed FGF23 homeostasis, the plasma levels of FGF23 do not reach the extreme levels as seen in CKD patients. Moreover, an impact on the renin–angiotensin–aldosterone system has also been proposed. FGF23 was shown to stimulate sodium reabsorption in the distal convoluted tubule and thereby increase circulating volume affecting the cardio-vascular system and the heart [116]. FGF23 has further been shown in experimental models to affect the immune system. It was demonstrated that FGF23 triggers the liver to produce inflammatory cytokines such as IL-6 and CRP via FGFR4 [117]. In another study it was shown that FGF23 weakens the host defense mechanism to infection and impairs leukocyte migration via FGFR2 [118]. CKD is known to be a state of inflammation and impairment of the immune system. Further studies are needed to clarify the clinical impact of these different complications to increased FGF23 in CKD patients. Similarly, the massive mortality of CKD patients due to the current COVID-19 pandemic deserves further investigation in respect to disturbed FGF23/Klotho system [119,120]. 

### 3.5. The Complex Interplay between FGF23 and PTH in Uremia

CKD patients suffering from severe kidney insufficiency have concomitant very high plasma levels of both hormones FGF23 and PTH. As such, the inhibitory effect of FGF23 on PTH secretion is abolished. In addition to the downregulation of CaSR, VDR and FGFR in the dysplastic parathyroid gland, it has been proposed to be explained by the downregulation of Klotho as shown by some groups [121,122,123]. However, the parathyroid expression of Klotho in CKD is very much model dependent and different studies found that the expression level of Klotho was related to the degree of kidney insufficiency and to varying expression within the gland [76,122,124]. Furthermore, the development of secondary hyperparathyroidism induced by renal failure did not differ in parathyroids specific klotho deletion mice compared to wildtype [57]. Collectively all studies illustrate a gradual change in pathophysiology to a more and more dysplastic autonomous secreting parathyroid gland in CKD progression.

Still, hypocalcemia is a common complication in kidney failure. Therefore, our findings of a superior role of the extracellular Ca^2+^ concentration in FGF23′s regulation of PTH, may illustrate an additional mechanism behind the resistance of the parathyroid gland to FGF23′s suppressive effect, as the concurrent low Ca^2+^ levels overrule this inhibition [20]. As previously mentioned, we demonstrated the inhibitory tonus which FGF23 executes on PTH secretion, when plasma calcium is within the normal range. This was also found in CKD rats with plasma Ca^2+^ within the normal range, as pharmacological inhibition of the FGFR resulted in increased PTH secretion [19]. Therefore, we speculate whether the increased FGF23 plasma levels in mild and moderate CKD actually have an inappropriate inhibitory effect on PTH secretion. This is adding another factor to the complex disturbance in the interplay between hormones in CKD-MBD. 

Whereas FGF23 impose a minute-to-minute regulation of PTH secretion, PTH stimulation of FGF23 expression is a slower stimulus. In uremia circulating PTH is necessary for generating the increase in FGF23 expression in bone and the high plasma levels of FGF23, as shown in experimental studies by us and others. Parathyroidectomy prevented the FGF23 increase in a CKD model. Additionally, it reduced FGF23 after CKD was inflicted in the rat [19,125]. In addition to kidney function, secondary hyperparathyroidism plays a superior role in the escalation of plasma levels of FGF23 in CKD. Yet, the precise mechanism is not known, as other stimulators of FGF23 in CKD are also present in the uremic milieu. New results on the intestine–bone axis determining the PTH effect on bone remodeling bring an additional level of regulation [126]. The impact of parathyroid disorders on FGF23 levels in CKD patients is unclear as conflicting results have been found in hemodialysis patients undergoing parathyroidectomy or treatment with calcimimetics [127,128,129].

## 4. New Factors Involved in CKD-MBD

In recent years, new factors have been proposed to be implicated in the disturbed mineral and bone disorder in CKD [130]. Kidney diseases reactivate development programs which are involved in nephrogenesis. For example, the Wnt pathway, which constitutes several Wnt ligands and Wnt inhibitors, is active in the developing kidney, but mostly silent afterwards. However, kidney injury induces the expression of Wnt and other factors. Whereas some only have autocrine/paracrine effects, others can be measured in higher levels in the circulation such as the Wnt inhibitors Dickkopf 1 (Dkk1), secreted frizzle-related proteins (SFRPs) and sclerostin as well as activin A that belongs to the TGF-β superfamily. These factors have specifically been proposed to be components of the CKD-MBD [130]. In bone, Wnt pathway continues to be active after embryogenesis and is an important anabolic pathway [37]. As such, these circulating Wnt inhibitors could potentially affect Wnt signaling in bone and impair bone formation. Wnt/ β-catenin has been proposed as a common pathogenic mediator of both heart and kidney lesions as shown in heart failure induced by transverse aorta constriction [131]. Klotho has protective role against both heart and kidney injury. The expression of renal Klotho is decreasing with the progression of kidney injury, where the amount of functional nephrons are reduced and the transfer of phosphate per nephron increased [132]. Interestingly, it has been suggested that the expression of renal Klotho was modulated by phosphaturia and that inhibition of Wnt/ β-catenin signaling prevented the phosphate induced downregulation of Klotho [133,134] 

Recently, we have examined such a new paradigm and its potential impact on CKD-MBD. We used the unilateral nephrectomy model (UUO), which is ideal to study the rapid development of unilateral renal fibrosis (characterized by induced periostin, decrease BMP7 and Klotho expression) in a non-uremic milieu as the contralateral kidney maintains a normal glomerular filtration [104]. Activin A was induced in the injured kidney after just one day of obstruction and its expression continued to increase. This was followed by a significant increase in plasma levels of activin A in circulation by day 10, secreted from the fibrotic kidney [34,104]. Another very interesting finding in this investigation was induction of sclerostin in the aorta, already at day 10 after UUO. So, the fibrotic kidney secretes factors that affect the vasculature even in the absence of a uremic state. Therefore, the contribution of the kidney to the disturbed system biology in CKD may take place before significant alterations in glomerular filtration [104]. Another experimental study has shown that activin A stimulates osteoclastogenesis via the RANK/RANKL system and IκBα-/NF-κB pathway [135]. Systemic activation of Activin receptors (ActR) in kidney, skeleton, vasculature and heart in CKD mouse models of diabetic nephropathy and Allport syndrome have been reported. Moreover, the treatment with the activin A binding protein follistatin or RAP-011 (a ligand trap of ActRIIA) has revealed amelioration of renal fibrosis and CKD-MBD findings in CKD models [136,137,138]. As such, activin A has been linked to uremic vasculopathy and renal osteodystrophy. Our group has demonstrated significant circadian rhythmicity of plasma activin A in normal rats with fourfold higher values in acrophase compared to nadir. The rhythmicity was severely disturbed in CKD rats [31]. 

In a study on atherosclerotic diabetic CKD mice, treatment with Dkk1 antibodies improved the renal osteodystrophy and uremic vasculopathy [139]. Treatment with sclerostin antibodies in a model of polycystic kidney disease showed an improved trabecular bone volume and cortical bone geometry, yet only when PTH levels were within the normal range [140]. Recently, it was shown that glycerol-3-phosphate secreted from the injured kidney increased FGF23 expression in bone [141]. Identification of these new factors in CKD-MBD is highly interesting and may in the future point toward therapeutic targets for patients with kidney disease. 

A pathological tissue crosstalk is a feature of CKD-MBD, and the disturbed system biology in CKD goes beyond impaired kidney function and kidney-derived factors. We proposed that vascular calcification in CKD could affect bone homeostasis, based on the findings of large upregulation of Wnt inhibitors and TGFβ family in the uremic vasculature. Among the upregulated signal molecules, we found potential circulating factors such as sclerostin, SFRP4 and activin A [77]. Recently, we demonstrated in vitro, a high secretion of sclerostin from incubated uremic calcified aorta rings [142]. As such, sclerostin is secreted by the calcified vasculature in CKD and can interfere with Wnt signaling in other organs. We transplanted normal rats with calcified aortas from CKD rats and examined the bone of recipients. The presence of the calcified aorta graft reduced bone mineral density and affected several pathways in bone [138]. So, the disturbances in the mineral and bone balance in CKD leading to soft tissue calcification is actually a vicious pathological crosstalk. Thus, the existence of a negative spiral of de-mineralization of bone and mineralization of the vasculature is demonstrated [142]. 

## 5. Summary

**Figure 4:**
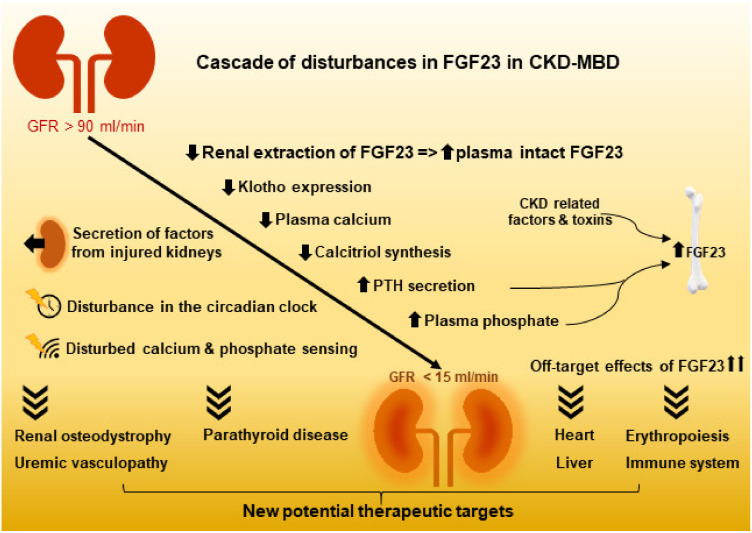
The loss of kidney function results not only in the complex disturbances in FGF23 and the mineral and bone homeostasis, but it also inflicts severe dysfunction of other organ systems. CKD: chronic kidney disease; CKD-MBD: CKD mineral and bone disorder.

Recent research has improved our understanding of the complex regulation of the hormones involved in mineral homeostasis. More local and circulating factors have been demonstrated to regulate FGF23 expression. Additionally, the extracellular concentration of Ca^2+^ affects the feed-back loop between FGF23 and PTH. How FGF23 is regulated by extracellular phosphate has not yet been completely clarified, but improvements have been made in understanding extracellular phosphate sensing. Thus, CaSR has binding sites for phosphate, where binding turns the receptor into an inactive state. Yet, how the two ligands Ca^2+^ and phosphate compete in CaSR activation/inactivation is not yet understood. In bone, Pit2 and FGFR1 has been proposed to be involved in phosphate sensing (Figure 4). 

A circadian rhythm of FGF23 has been demonstrated, yet the mechanism leading to the diurnal variation in plasma levels is not fully understood, although they are related to feeding. A molecular circadian clock is active in the parathyroid gland but gets disturbed in CKD. Additionally, the plasma levels of activin A and phosphate changed their diurnal pattern in CKD. These findings are important when interpreting biochemistry results in CKD patients. Still, the clinical importance of a disturbed circadian rhythmicity in CKD-MBD has yet to be finally established, so has its implications in the development of parathyroid hyperplasia. 

The kidney has long been known for its important role in the balance of calcium and phosphate by regulating Ca^2+^ and phosphate reabsorption in the kidney and the plasma levels of calcitriol. In addition, the key role of the kidney in regulating the plasma levels of intact FGF23 has been identified by a high extraction ratio of 40% between the renal artery and vein. This important degradation of the hormone diminishes in CKD, resulting in increased plasma levels. Expression of FGF23 is induced in injured kidney and probably has a role in the inflammation and fibrosis processes via autocrine/paracrine signaling. Kidney-derived FGF23 does not contribute to the increased plasma levels of FGF23 in CKD. 

Recently, new factors related to Wnt or TGF-β signaling have been shown to be secreted from the injured kidney and the uremic calcified vasculature, and they may have an important role in CKD-MBD. Future studies may further provide understanding of each of these factors and their potential as therapeutic targets in CKD and CKD-MBD.
